# Supplementary material for: Tead1a Initiates Transcriptional Priming Through the TEAD1a/YAP‐Notch1‐Spi1/Cebpα Axis to Promote Neutrophil Fate
Source: Adv Sci (Weinh). 2025 Aug 28;12(41):e05441. doi: 10.1002/advs.202505441 (PMC12591127; doi:10.1002/advs.202505441)
Supplement: Supplementary file 4 — Supplementary Dataset [file ADVS-12-e05441-s004.zip › 3-6.zip!/3-6/02-6.cluster/cluster15/Enrich/ego_all.pdf]

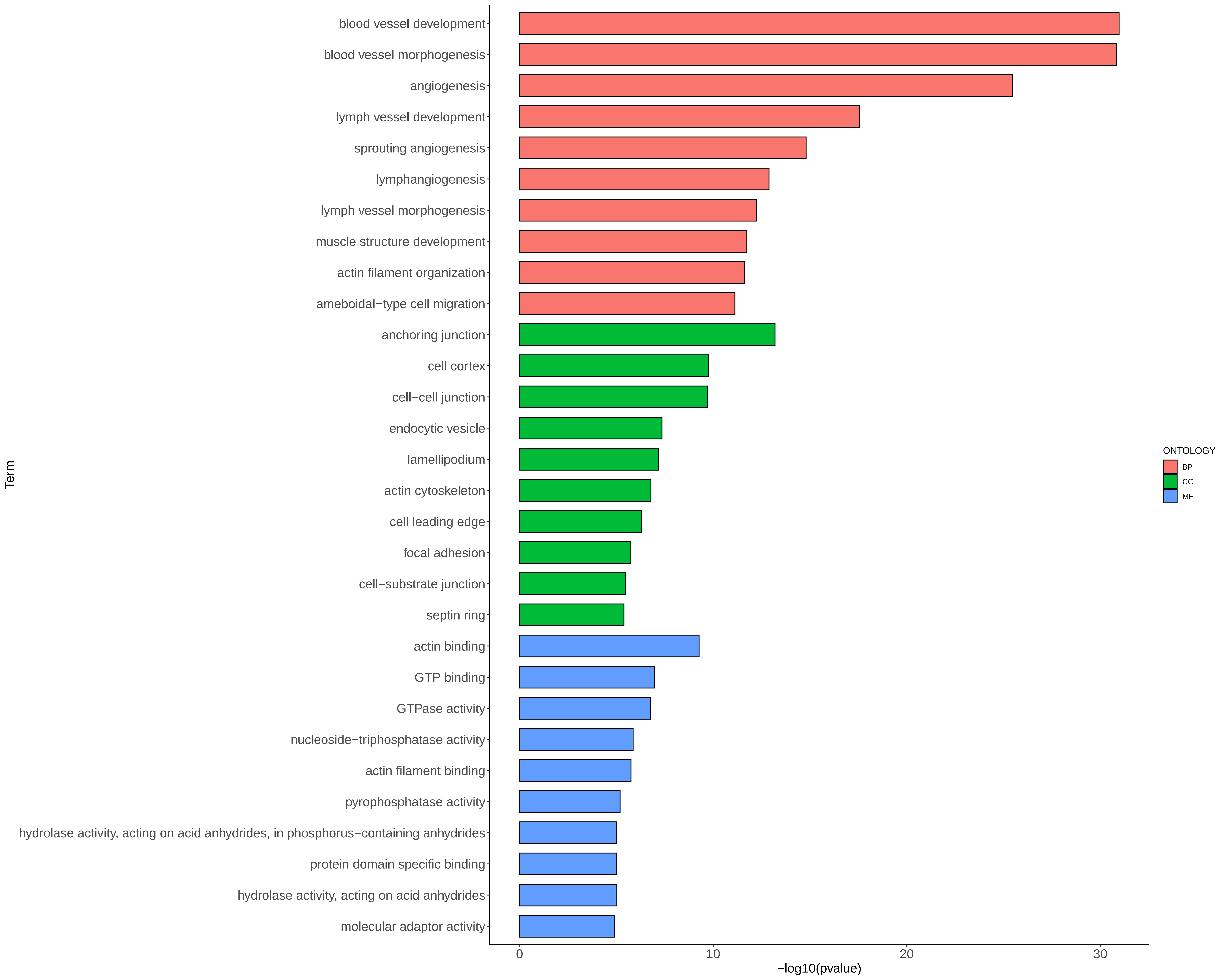
blood vessel development
blood vessel morphogenesis
angiogenesis
lymph vessel development
sprouting angiogenesis
lymphangiogenesis
lymph vessel morphogenesis
muscle structure development
actin filament organization
ameboidal−type cell migration
anchoring junction
cell cortex
cell−cell junction
endocytic vesicle
lamellipodium
actin cytoskeleton
cell leading edge
focal adhesion
cell−substrate junction
septin ring
actin binding
GTP binding
GTPase activity
nucleoside−triphosphatase activity
actin filament binding
pyrophosphatase activity
hydrolase activity, acting on acid anhydrides, in phosphorus−containing anhydrides
protein domain specific binding
hydrolase activity, acting on acid anhydrides
molecular adaptor activity
Term
0
10
20
30
−log10(pvalue)
ONTOLOGY
BP
CC
MF
